# Quantitative and Qualitative Evaluation of a Confidence-Aware Transformer-Based Super-Resolution Framework for Panoramic Radiographs

**DOI:** 10.1016/j.identj.2026.109590

**Published:** 2026-04-27

**Authors:** Jaehyup Lee, Chang-Hyeon An, Seo-Young An, Eun-Kyong Kim, Young-Eun Kwon

**Affiliations:** a Department of Computer Science and Engineering, Kyungpook National University, Daegu, Republic of Korea; b Department of Oral and Maxillofacial Radiology, Kyungpook National University School of Dentistry, IHBR, Daegu, Republic of Korea; c Department of Oral and Maxillofacial Radiology, Kyungpook National University School of Dentistry, IHBR, ITRD, Daegu, Republic of Korea; d Department of Preventive Dentistry, School of Dentistry, Kyungpook National University, Daegu Republic of Korea

**Keywords:** Panoramic radiography, Deep learning, Image processing, Computer-assisted, Artificial intelligence, Diagnostic imaging

## Abstract

**Objectives:**

This study aimed to develop and evaluate a confidence-aware transformer-based super-resolution framework, termed CAT-PRSR, to enhance image quality and diagnostic reliability in panoramic dental radiographs.

**Methods:**

A total of 1078 anonymised panoramic radiographs were retrospectively collected (950 for training, 128 for testing). The CAT-PRSR framework integrating a transformer-based SR backbone with a confidence-aware training strategy was developed. The model generates a high-resolution output with pixel-wise uncertainty estimation, allowing adaptive learning focused on diagnostically relevant regions while minimising over-enhancement in noise-sensitive areas. Model performance was evaluated using 6 quantitative metrics – peak signal-to-noise ratio (PSNR), structural similarity index (SSIM), spatial correlation coefficient (SCC), natural image quality evaluator (NIQE), learned perceptual image patch similarity (LPIPS), and Fréchet inception distance (FID)—and mean opinion score (MOS) assessment. Based on quantitative performance, 4 representative state-of-the-art SR models were selected for comparison at 4×, 6×, and 8× magnifications.

**Results:**

CAT-PRSR demonstrated superior performance across all metrics and magnification levels. It achieved the highest peak signal-to-noise ratio (36.41 at 4 ×, 36.19 at 6 ×, and 33.73 at 8 ×) and the lowest FID (1.77, 9.29, and 2.09, respectively), outperforming all comparison models. In MOS evaluations, CAT-PRSR maintained diagnostic utility scores statistically comparable to ground truth images (*P* > .05), while other models showed significant degradation (*P* < .001).

**Conclusion:**

The proposed CAT-PRSR framework demonstrated potential to enhance panoramic radiograph resolution by integrating pixel-level fidelity with improved diagnostic reliability.

**Clinical Relevance:**

The CAT-PRSR model may enhance the diagnostic reliability of panoramic radiographs acquired under low-resolution conditions, supporting more accurate clinical decision-making and serving as a reliable imaging resource for AI-driven dental research.

## Introduction

Panoramic radiography (PR) is a fundamental diagnostic imaging modality in dentistry, providing a comprehensive two-dimensional overview of the maxillofacial region, including the maxilla, mandible, dentition and adjacent anatomical structures.^1^^,^^2^ Despite its broad coverage and clinical utility, PR inherently suffers from limited spatial resolution, geometric distortion and image blurring caused by the curved acquisition geometry, patient positioning errors and motion artifacts.^1^^,^^3^^,^^4^ These limitations often obscure fine anatomical details, making it challenging to identify small lesions or subtle structural abnormalities, which may compromise diagnostic accuracy and increase the risk of misdiagnosis.^3^ In recent years, the rise of artificial intelligence (AI) – especially deep learning – in dentistry has demonstrated remarkable success in automatically detecting pathologies (eg, caries, periodontal bone loss, periapical lesions, tumours) from radiographs.^2^, ^3^, ^4^, ^5^ These AI models perform best with high-quality inputs; thus, enhancing the resolution and overall image quality of panoramic radiographs represents a critical step toward improving both clinician-driven and AI-assisted diagnostic performance.^1^^,^^3^^,^^5^^,^^6^

Super-Resolution (SR) aims to reconstruct high-resolution (HR) images from low-resolution (LR) inputs, thereby enhancing anatomical detail and overall image quality.^1^^,^^3^^,^^5^, ^6^, ^7^, ^8^, ^9^ Originating from traditional interpolation-based upscaling methods such as bilinear and bicubic interpolation, SR study has evolved toward learning-based approaches that can recover high-frequency information rather than merely estimating pixel intensity.^1^^,^^3^^,^^9^^,^^10^ The development of deep learning marked a turning point in SR advancement.^3^^,^^5^^,^^7^^,^^9^ The Super-Resolution Convolutional Neural Network (SRCNN) first introduced an end-to-end learning framework for LR-to-HR mapping, followed by Generative Adversarial Networks (GANs), which achieved perceptually realistic image restoration through adversarial training.^1^^,^^3^^,^^7^^,^^9^^,^^11^ Most recently, transformer-based architectures – leveraging self-attention mechanisms to capture long-range dependencies and global context – have demonstrated superior performance in various medical imaging tasks, including SR.^6^^,^^12^ SR in PR remains limited, despite growing SR literature in dental and medical imaging.^1^^,^^5^^,^^8^^,^^11^^,^^13^ Moran et al.^8^ successfully applied SRGAN with transfer learning to improve the resolution and diagnostic quality of periapical radiographs. Comparative analyses of multiple SR models (eg, SRCNN, SRGAN, U-Net, SwinIR and LTE) on panoramic radiographs have shown that deep learning–based SR consistently enhances image fidelity.^1^ Furthermore, a recent multicentre study demonstrated that incorporating SR preprocessing improved the predictive performance of AI models for assessing mandibular third molar extraction difficulty.^5^

However, most existing SR models apply uniform enhancement across the entire image, disregarding the varying diagnostic importance of different anatomical regions. This can result in over-processing of noise-prone areas and insufficient enhancement of clinically critical structures.^1^^,^^3^^,^^8^^,^^12^ To address these limitations, this study proposes CAT-PRSR (Confidence-Aware Transformer-based Panoramic Radiography Super-Resolution), a novel framework that integrates pixel-wise uncertainty modelling with transformer-based spatial encoding to adaptively emphasise diagnostically relevant regions while suppressing noise, thereby improving both interpretability and diagnostic reliability.

This study aimed to determine whether the proposed CAT-PRSR framework could enhance image quality and improve diagnostic reliability of panoramic radiographs compared with existing state-of-the-art SR models.

## Materials and methods

### Dataset

A total of 1078 anonymised digital panoramic radiographs were retrospectively collected from the Picture Archiving and Communication System (PACS) of Kyungpook National University Dental Hospital and divided into 950 training and 128 testing images. Radiographs were acquired between January 2015 and December 2024 using 1 of 2 panoramic X-ray units (Orthopantomograph OP100D or OP200D; Instrumentarium Imaging). Images showing motion artifacts, detector errors, or truncated fields of view were excluded according to predefined criteria. All eligible images were exported in TIFF format (2972 × 1536 pixels) to preserve spatial fidelity and anonymised by removing all patient identifiers. This retrospective study was approved by the Institutional Review Board of Kyungpook National University Dental Hospital (IRB No. KNUDH-2025-02-03-00).

### Model architectures

**Fig. 1:**
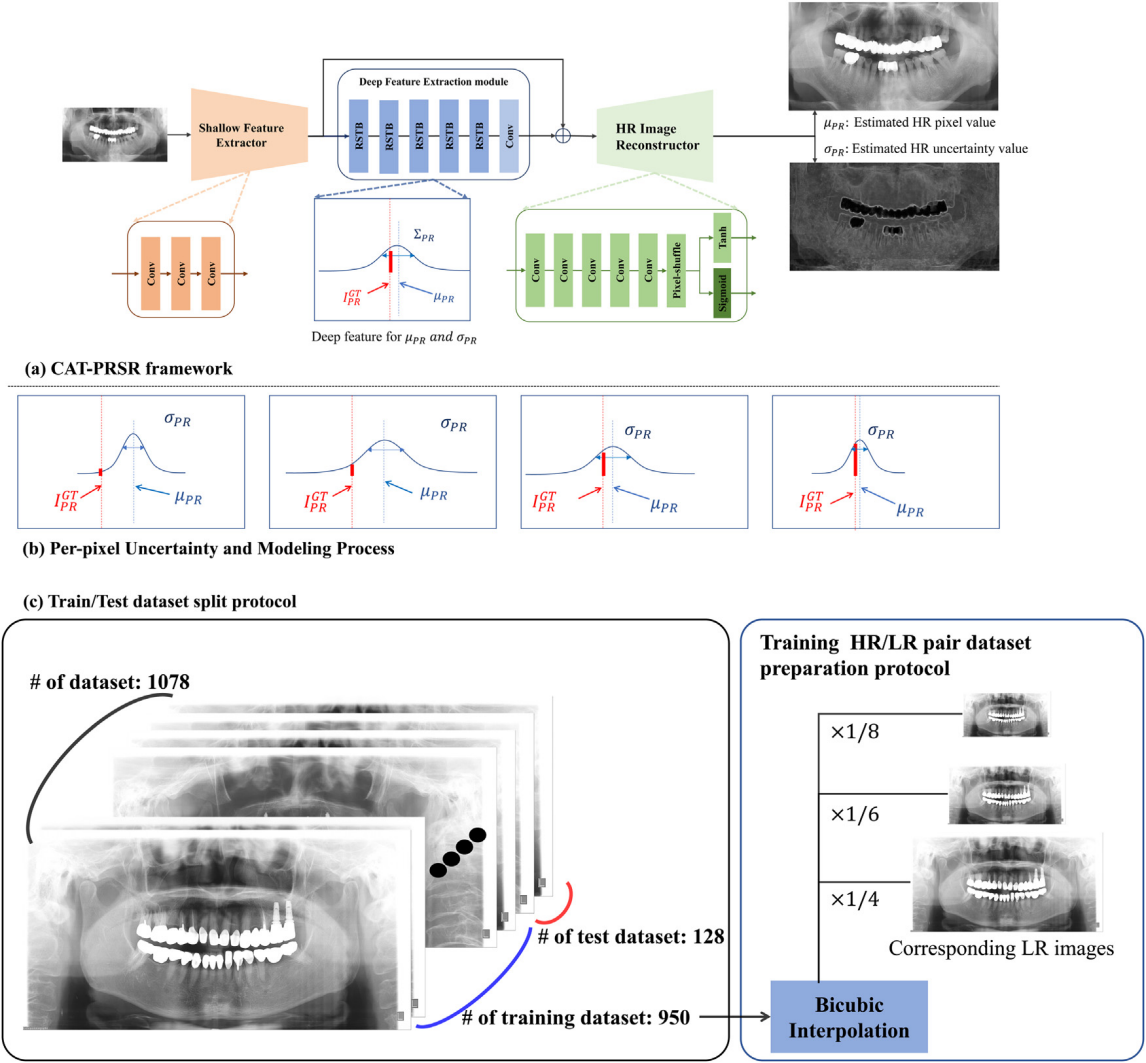
Overview of the CAT-PRSR framework and dataset preparation. (A) Architecture of CAT-PRSR, comprising a shallow feature extractor, residual Swin transformer blocks (RSTB), and a dual-head output predicting high resolution (HR) image and uncertainty. (B) Uncertainty-guided loss process based on per-pixel Laplace modelling. (C) Dataset preparation and split protocol: 1078 panoramic images (950 training, 128 test) with low-resolution (LR)–high-resolution (HR) pairs generated via bicubic interpolation at × 1/4, × 1/6, and × 1/8 scales.

The CAT-PRSR network is designed to reconstruct HR panoramic images from LR inputs while simultaneously estimating the uncertainty associated with each reconstructed pixel (Figure 1A). Given a panoramic input image $I_{LR}\in R^{H\times W\times \;1}$, the network jointly predicts 2 complementary outputs: an SR image $\mu_{PR}\in R^{\left(4H)\;\times \;(4W\right)}$and a corresponding pixel-wise uncertainty map $\sigma_{PR}\in R^{\left(4H)\;\times \;(4W\right)}$. This dual-output design enables both accurate reconstruction and reliable quantification of confidence, which is particularly critical in medical panoramic imaging where visual consistency and interpretability are essential. While retaining the proven RSTB backbone of SwinIR for efficient long-range dependency modelling, CAT-PRSR introduces pixel-wise Laplace uncertainty estimation and dual-head reconstruction as its primary innovations. The architecture is composed of 4 primary components: (1) a shallow feature extractor, (2) a deep feature extractor built upon Residual Swin Transformer Blocks (RSTBs), (3) a residual skip connection for feature fusion and (4) a dual-head reconstruction module that outputs both intensity and uncertainty predictions. The detailed and reproducible backbone architecture specifications, including the number of RSTBs, transformer layers, window size, embedding dimension, attention heads and total parameter count, are provided in Table S1.

#### Shallow feature extraction

In the initial stage, the low-resolution input image $I_{LR}$passes through a series of 3 two-dimensional convolutional layers. These layers operate as a shallow feature extractor that captures fundamental image characteristics, such as local edges, gradient transitions and fine textural cues. By employing progressively larger receptive fields and nonlinear activations, this stage transforms the raw intensity input into a compact yet informative feature representation. These extracted shallow features, denoted as $F_{shallow}$, act as the foundational representation upon which deeper, contextually richer features are built in subsequent transformer-based stages. This shallow stage ensures that low-level structural integrity, crucial for anatomical consistency in medical panoramas, is preserved throughout the network.

#### Deep feature extraction via residual Swin transformer blocks (RSTBs)

The deep feature extraction backbone of CAT-PRSR is constructed from a sequence of RSTBs. Each RSTB integrates window-based self-attention mechanisms, local positional encodings and gated feed-forward networks. By partitioning the feature space into nonoverlapping local windows and applying self-attention within these regions, the model efficiently captures fine-grained spatial dependencies while maintaining computational tractability. Furthermore, through shifted window mechanisms, inter-window information exchange is facilitated, allowing the network to perceive long-range global dependencies that are particularly significant in panoramic imaging, where clinically relevant structures can span extensive spatial extents. Each RSTB outputs a refined feature representation $F_{deep}$, enriched with both global contextual awareness and local structural fidelity. The residual connections embedded within each transformer block promote stable gradient flow and encourage effective feature reuse, thereby enhancing convergence stability during training.

#### Residual skip connection for feature fusion

To balance low- and high-frequency feature representations, CAT-PRSR introduces a residual skip connection that directly links the shallow feature extractor to the deep feature extraction module. This connection allows the network to fuse fine low-frequency spatial information from $F_{shallow}$with the high-level contextual representations from $F_{deep}$, producing a combined feature map $F_{fusion}$. Such skip connections mitigate potential information loss that may arise during deep hierarchical transformations, ensuring that the reconstruction preserves both the global luminance consistency and the fine anatomical contours. Moreover, they enhance gradient propagation during backpropagation, facilitating efficient optimisation and preventing degradation in deeper layers.

#### Dual-head reconstruction and pixel shuffle upsampling

The fused feature representation $F_{fusion}$is subsequently processed through a series of convolutional layers followed by a Pixel Shuffle upsampling operation. The Pixel Shuffle module rearranges feature channels into spatial dimensions, thereby increasing the resolution by a factor of 4 in both height and width. This operation is computationally efficient and maintains local spatial coherence during the upsampling process. After upsampling, the resulting high-resolution feature map branches into 2 distinct heads, each specialised for a different prediction target:

1. *Image reconstruction head:* This head is responsible for generating the final super-resolved output image $\mu_{PR}$. It consists of a sequence of convolutional layers followed by a Tanh activation function, which constrains the output pixel intensities to the range [−1,1]. The use of Tanh ensures stable output normalisation and smooth gradient behaviour, effectively capturing subtle tonal variations while maintaining sharp boundaries in reconstructed anatomical structures.
2. *Uncertainty estimation head:* The second branch predicts the corresponding pixel-wise uncertainty map $\sigma_{PR}$, which represents the logarithmic scale parameter of the predicted Laplace distribution. This branch concludes with a Sigmoid activation function, ensuring that uncertainty values are bounded within [0,1], thus providing an interpretable and normalised confidence map for each pixel. Higher values in $\sigma_{PR}$ correspond to greater predictive uncertainty, guiding downstream tasks such as reliability-weighted training and diagnostic confidence assessment.

#### Probabilistic modelling

Each reconstructed pixel is modelled as a random variable following a Laplace distribution parameterised by the predicted mean $\mu_{PR}\left(x,y\right)$and scale (uncertainty) $\sigma_{PR}\left(x,y\right)$:

$$p\left(I_{HR}\left(x,y\right)|I_{LR}\right)=\frac{1}{2\sigma_{PR}\left(x,y\right)}\text{exp}\left(-\frac{\left|I_{HR}\left(x,y\right)-\mu_{PR}\left(x,y\right)\right|}{\sigma_{PR}\left(x,y\right)}\right)$$

This probabilistic formulation allows CAT-PRSR to not only reconstruct high-frequency details but also quantify the reliability of each prediction. During training, the network optimises a negative log-likelihood (NLL) loss derived from the Laplace distribution, which is mathematically equivalent to a weighted $L_{1}$ loss function (Figure 1B). The Laplace model, characterised by heavier tails compared to the Gaussian, is particularly advantageous in medical imaging because it is more robust to outliers and better preserves discontinuities at tissue boundaries or sharp anatomical interfaces. Moreover, by dynamically adjusting its uncertainty estimates, CAT-PRSR can down-weight unreliable or noisy regions during optimisation, effectively focusing learning on spatially stable regions while maintaining resilience against localised noise or structural inconsistencies. Importantly, the predicted pixel-wise uncertainty is not treated as an auxiliary output but is directly incorporated into the optimisation process through the NLL loss. This uncertainty-guided weighting allows the network to adaptively modulate learning across spatial regions, reducing the influence of unreliable or noise-dominated pixels during training.

#### Experimental design

A 2-stage evaluation framework was employed to assess SR models on panoramic radiographs, balancing thoroughness with computational efficiency (Figure 1C). Stage 1 served as a rapid screening step to identify the most promising algorithms based on performance at a single magnification (4×). Eight representative SR models – SRGAN,^13^ VDSR,^14^ EDSR,^15^ SwinIR,^16^ RealESRGAN,^17^ RCAN,^18^ SeD,^19^ and the proposed CAT-PRSR – were evaluated using standardised panoramic datasets. Stage 2 performed an in-depth evaluation on the 3 top-performing models from Stage 1 (RealESRGAN,^17^ SwinIR^16^ and SeD^19^) together with CAT-PRSR across multiple magnification factors (4×, 6× and 8×). This stage comprehensively examined reconstruction fidelity, perceptual realism and expert-based diagnostic quality under varying upsampling conditions. All models were trained under identical optimisation settings to ensure fair comparison across architectures. Training loss was continuously monitored, and all models exhibited stable convergence over the course of training.

#### Training protocol

All super-resolution models, including the proposed CAT-PRSR and baseline comparators, were implemented using the PyTorch deep learning framework and trained on a workstation with an NVIDIA RTX 4090 GPU, AMD Ryzen 7900 CPU and 64 GB RAM. To ensure stable optimisation and convergence, the AdamW optimizer (initial learning rate of $1\;\times 10^{-4}$, weight decay $1\;\times 10^{-7}$) was adopted. Each model was trained for a total of 1,000,000 iterations, with both the learning rate and weight decay reduced by a factor of 10 after 500,000 iterations. The mini-batch size was fixed at 4 for all experiments to balance computational efficiency and convergence stability. To construct paired training data, HR panoramic radiographs were downsamplled using bicubic interpolation to generate low-resolution LR counterparts. The downsamplling was performed at 3 magnification factors 4×, 6× and 8×, to create LR images with different degradation levels. Each LR image was paired with its original HR image to form a dataset specific to each magnification scale, facilitating scale-dependent learning. During training, a patch-wise strategy was employed to improve data diversity and optimise memory efficiency. For the 4× scale, HR and LR patch sizes were set to 128×128 and 32×32, respectively. For the 6× and 8× scales, HR patch sizes were increased to 192×192 and 256×256, while the LR patch size was consistently maintained at 32×32 to preserve uniform receptive field coverage across scales. This multiscale patch configuration enabled localised feature learning suitable for both coarse and fine details while maintaining stable training dynamics. All preprocessing steps, including normalisation and data augmentation, were applied uniformly to both training and test datasets to ensure experimental consistency. Throughout training, the models learned to reconstruct high-fidelity HR patches from corresponding LR inputs, effectively capturing fine structural and textural features in panoramic images. Figure 1 provides an overview of the training pipeline and data preparation process.

#### Quantitative evaluation metrics

Quantitative evaluation was conducted using 4 widely adopted image quality metrics: Peak Signal-to-Noise Ratio (PSNR), Structural Similarity Index Measure (SSIM), Spatial Correlation Coefficient (SCC) and Natural Image Quality Evaluator (NIQE), Learned Perceptual Image Patch Similarity (LPIPS) and Fréchet Inception Distance (FID). Together, these metrics comprehensively assess pixel-level fidelity, perceptual realism and distributional similarity in reconstructed panoramic radiographs.

(1) PSNR and SSIM

PSNR quantifies pixel-level accuracy by comparing the maximum possible pixel value of an image with the mean squared error (MSE) between the reconstructed image $\hat{I}$ and the ground truth I. It is defined as:

$$\text{PSNR}=10\cdot \log_{10}\left(\frac{MAX^{2}}{\frac{1}{N}\sum_{i=1}^{N}{\left(I_{i}-{\hat{I}}_{i}\right)}^{2}}\right)$$

Where MAX is the maximum pixel value (eg, 255), and N is the number of pixels.

The SSIM evaluates perceptual similarity between 2 images by jointly measuring luminance, contrast and structural consistency:

$$\text{SSIM}\left(x,y\right)=\frac{\left(2\mu_{x}\mu_{y}+c_{1}\right)\left(2\sigma_{xy}+c_{2}\right)}{\left({\mu_{x}}^{2}+{\mu_{y}}^{2}+c_{1}\right)+\left({\sigma_{x}}^{2}+{\sigma_{y}}^{2}+c_{2}\right)}$$

Where $\mu_{x}$and $\mu_{y}$are the means, ${\sigma_{x}}^{2}$and ${\sigma_{y}}^{2}$are the variances, and $\sigma_{xy}$ is the covariance between the output image x and the reference image y. The constants $c_{1}$ and $c_{2}\;$are used to stabilise the division with weak denominators.

(2) SCC and NIQE

SCC evaluates spatial correlation and geometric alignment between reconstructed and reference images, providing a measure of structural consistency – particularly relevant in dental imaging where edge preservation is critical. NIQE is a no-reference metric that estimates perceptual naturalness without requiring ground-truth data. Lower NIQE values indicate fewer artifacts and a more natural appearance, making it useful when HR references are unavailable.

(3) LPIPS and FID

LPIPS measures perceptual similarity by computing deep feature distances between 2 images using pretrained neural networks such as AlexNet or VGG.

$$\text{LPIPS}=\;\sum_{l}\frac{1}{H_{l}W_{l}}\sum_{h,w}\left\|w_{l}⊙\left(\emptyset_{l}{\left(x\right)}_{h,w}-\emptyset_{l}{\left(y\right)}_{h,w}\right)\right\|\;$$

where, $\emptyset_{l}\left(\cdot \right)\in R^{H_{l}\times W_{l}\times C_{l}}\;$denotes the feature map extracted from the l-th layer of the pretrained network, and $w_{l}\in R^{C_{l}}$ represents a set of learned channel-wise weights used to calibrate the relative importance of feature channels. The operator ⊙ denotes element-wise multiplication. This learned weighting scheme enables LPIPS to better reflect human perceptual judgment by emphasising salient features and reducing less informative ones. Lower LPIPS scores indicate greater perceptual similarity and more faithful texture and contrast reproduction than pixel-based metrics such as PSNR and SSIM.

The FID measures the distance between feature distributions of real and reconstructed images in the latent space of an Inception-V3 network. The metric is defined as:

$$\text{FID}={∥\mu_{r}-\mu_{g}∥}_{2}^{2}+\text{Tr}\left(\Sigma_{r}+\Sigma_{g}-2{\left(\Sigma_{r}\Sigma_{g}\right)}^{1/2}\right)$$

where $(\mu_{r},\Sigma_{r})\;$and $\left(\mu_{g},\Sigma_{g}\right)$are the mean and covariance of feature embeddings for real and generated images, respectively. A lower FID indicates that the reconstructed images have feature distributions closer to those of real images, reflecting higher perceptual and generative quality.

### Ablation setting (w/o uncertainty)

To isolate the contribution of uncertainty estimation, we additionally evaluated a deterministic variant of our model, denoted as CAT-PRSR (w/o uncertainty). In this variant, the architecture and all hyperparameters are identical to CAT-PRSR, except that the uncertainty estimation branch is removed at the final output stage. Concretely, after the shared backbone and PixelShuffle upsampling, the network outputs only the super-resolved intensity image $\mu_{PR}$​ (single output channel) and does not predict the per-pixel uncertainty map $\sigma_{PR}$. The model is trained using the standard pixel-wise reconstruction loss without uncertainty-guided weighting. It was trained using a standard pixel-wise $L_{1}$ loss (ie, without the Laplace NLL weighting term).

### Subjective evaluation (mean opinion score analysis)

For qualitative image-quality assessment, mean opinion scores (MOS) were obtained from 3 board-certified oral and maxillofacial radiologists (each with >10 years of experience). Each expert independently evaluated images reconstructed by 4 SR models (SRGAN, SwinIR, SeD and CAT-PRSR) and their corresponding ground truths (GT). Each image was rated on a 5-point Likert scale (1 = poor, 5 = excellent) across 6 diagnostic criteria: (1) Overall image quality, (2) anatomical visibility, (3) boundary sharpness, (4) microstructure visibility, (5) noise/artifact suppression and (6) diagnostic utility.

### Statistical analysis

All statistical analyses were performed in Python 3.11.6 (pandas 2.3.3, NumPy 2.3.4) and figures were created using Matplotlib 3.8.1. Mean ± standard deviation (SD) values were reported for each observer and image type. Statistical differences between each SR model and GT were analysed using the paired t-test for each observer. The significance threshold was set at *P < .05*. Asterisks in the tables indicate statistical significance levels (*P < .05, P < .01, P < .001*), while '*ns*' denotes nonsignificant differences. All analyses were performed separately for 6 × and 8 × magnification factors. For the qualitative assessment using MOS, inter-observer agreement was evaluated descriptively using percent agreement, including both exact agreement and agreement within ±1 point, across all evaluation metrics for each model and the ground truth. This approach was adopted to provide a clinically interpretable measure of concordance for the ordinal 1 to 5 MOS scale.

## Results

### Preliminary model screening (stage 1)

To systematically identify the most promising SR models for comprehensive evaluation, 8 state-of-the-art approaches were compared using 4 quantitative metrics (Table 1, Figure S1). Among all evaluated models, CAT-PRSR demonstrated the highest overall performance, achieving a PSNR of 42.01, SSIM 0.950, SCC 0.998 and the lowest NIQE 2.439 – indicating superior pixel fidelity, structural alignment and perceptual realism. The next best models, SeD and SwinIR, achieved PSNR values around 40.9 and 40.1, respectively, with higher NIQE scores (2.605-3.209), showing slightly reduced perceptual quality. Conversely, RCAN showed a relatively high NIQE (4.761) despite moderate reconstruction fidelity, suggesting perceptual over-sharpening. Based on Stage 1, RealESRGAN, SwinIR and SeD were selected as representative baselines for Stage 2 in-depth evaluation due to their balanced performance across fidelity and perceptual metrics.

**Table 1:** Quantitative comparison of 8 super-resolution models across 4 evaluation metrics.

	SRGAN	VDSR	EDSR	SwinIR	RealESRGAN	RGAN	SeD	CAT-PRSR
PSNR↑	39.93	40.47	40.81	40.07	39.72	40.14	40.92	42.01
SSIM↑	0.923	0.882	0.930	0.942	0.941	0.944	0.943	0.950
SCC↑	0.892	0.905	0.988	0.982	0.974	0.969	0.984	0.998
NIQE↓	3.502	3.519	3.410	3.209	2.890	4.761	2.605	2.439

Evaluation metrics: PSNR = Peak Signal-to Noise Ratio, SSIM = Structural Similarity Index Measure, SCC = Spatial Correlation Coefficient, NIQE = Natural Image Quality Evaluator (lower is better).

### Quantitative performance across magnification factors (stage 2)

**Fig. 2:**
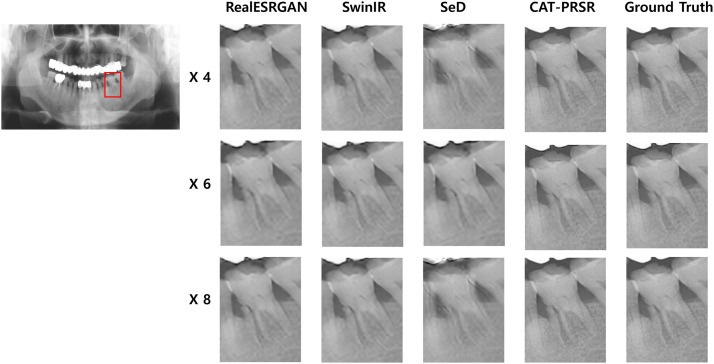
Model comparison across magnification scales (Stage2). Cropped panoramic regions reconstructed by RealESRGAN, SwinIR, SeD, and CAT-PRSR at 4 ×, 6 ×, and 8 × magnifications.

**Fig. 3:**
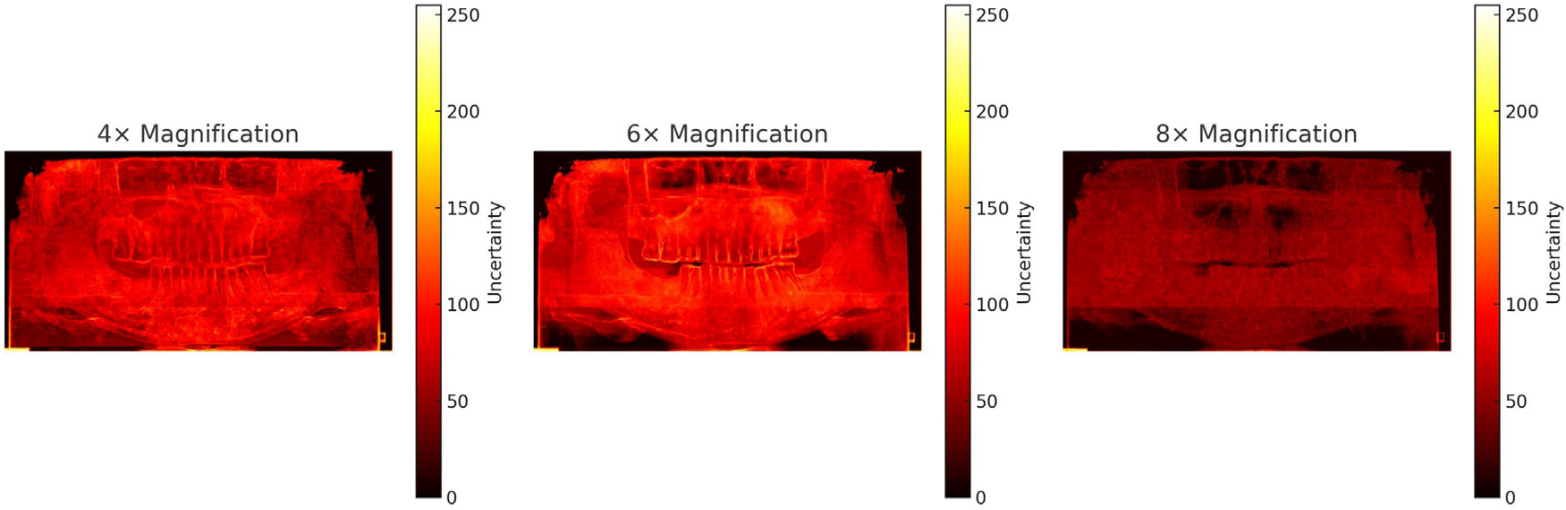
Heatmap visualization of model uncertainty across magnification scales. Uncertainty maps of CAT-PRSR outputs at 4 ×, 6 ×, and 8 × magnifications. Uncertainty values are spatially relative, illustrating qualitative patterns of spatial consistency and confidence stability rather than absolute scale-to-scale comparison. Colour bars indicate normalized uncertainty from 0 (low) to 255 (high).

Based on Stage 1 results, Stage 2 conducted a multiscale comparison (4×, 6× and 8×) among the 3 top-performing baselines – RealESRGAN, SwinIR and SeD – and the proposed CAT-PRSR. Across all magnification levels (4×, 6× and 8×), CAT-PRSR consistently outperformed competing models in both pixel-wise fidelity and perceptual realism (Table 2, Figure 2). At 4× magnification, CAT-PRSR reached the highest PSNR (36.41) and SSIM (0.9996), with the lowest LPIPS (0.2177) and FID (1.777) – surpassing RealESRGAN (33.75, 0.9984) and SwinIR (32.73, 0.9972). At 6×, CAT-PRSR maintained a clear advantage (PSNR 36.19, SSIM 0.9984) while reducing perceptual deviation (FID 9.29 vs 46-59 for others). At 8×, the performance gap widened: CAT-PRSR achieved PSNR 33.73 and SSIM 0.9985, with substantially lower FID (2.09) compared to SeD (158.6) and SwinIR (39.1). As shown in Table 2, incorporating uncertainty prediction enables uncertainty-guided learning and yields consistent gains in both reconstruction fidelity (PSNR/SSIM) and perceptual metrics (LPIPS/FID) across all scales. Figure 3 illustrates the proposed model’s spatial uncertainty distributions across different magnification levels. These uncertainty maps are presented for qualitative interpretation only, providing insight into spatial consistency and confidence stability of the CAT-PRSR outputs. As uncertainty values are spatially relative, the visualisations qualitatively illustrate that the model maintains stable uncertainty patterns and consistent structural reliability across magnification levels rather than allowing direct magnitude comparison.

**Table 2:** Quantitative comparison of 4 super-resolution models by Magnification Scale.

		PSNR↑	SSIM↑	LPIPS ↓	FID ↓
×4	RealESRGAN	33.75	0.9984	0.3443	2.294
	SwinIR	32.73	0.9972	0.3498	4.045
	SeD	32.62	0.9972	0.3504	4.388
	CAT-PRSR	36.41	0.9996	0.2177	1.777
	CAT-PRSR (w/o uncertainty)	32.65	0.9970	0.3520	4.612
×6	RealESRGAN	35.17	0.9798	0.4962	46.13
	SwinIR	35.3	0.9799	0.5009	59.14
	SeD	35.27	0.98	0.4972	47.15
	CAT-PRSR	36.19	0.9984	0.4216	9.293
	CAT-PRSR (w/o uncertainty)	35.22	0.9792	0.5031	59.82
×8	RealESRGAN	32.56	0.984	0.4722	29.84
	SwinIR	32.51	0.9841	0.4762	39.11
	SeD	30.73	0.9615	0.5086	158.6
	CAT-PRSR	33.73	0.9985	0.3439	2.093
	CAT-PRSR (w/o uncertainty)	32.43	0.9839	0.4785	41.85

Quantitative evaluation of SR models at × 4, × 6, and × 8 upscaling. Bold indicates best performance per metric (↑ higher is better, ↓ lower is better).

### Qualitative assessment by MOS (stage 2)

**Fig. 4:**
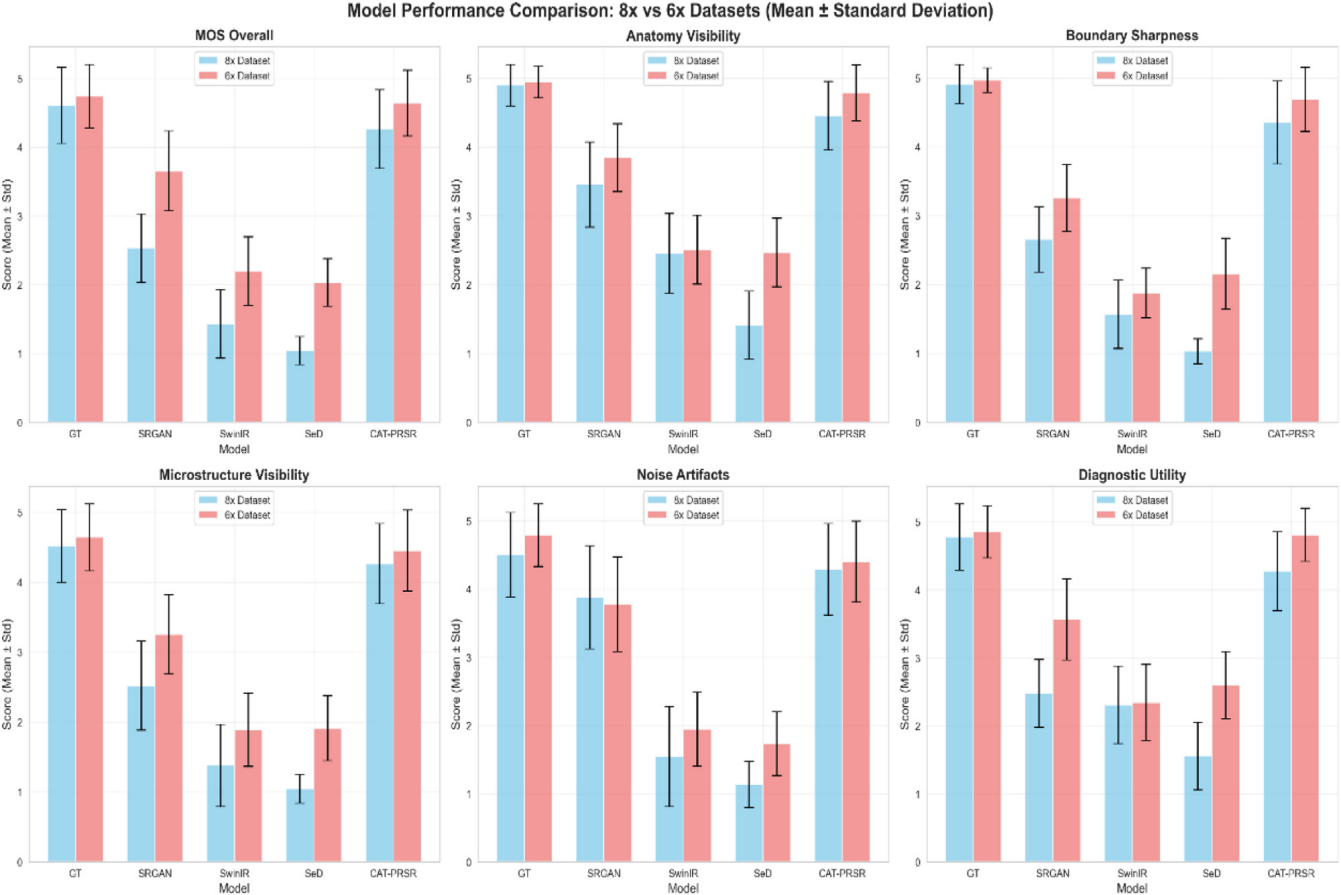
MOS-based comparison at 6× and 8× magnifications. Mean Opinion Scores (MOS) across 6 qualitative criteria—overall quality, anatomy visibility, boundary sharpness, microstructure visibility, noise suppression, and diagnostic utility—for each SR model. CAT-PRSR achieved the highest scores, closely matching the ground truth (GT) across all evaluation categories.

Expert evaluations revealed that CAT-PRSR consistently achieved high perceptual and diagnostic quality at both 6× and 8× magnification levels (Figure 4). Detailed MOS scores for each observer and criterion are provided in Supplementary Table S2. Perfect agreement (exact score match) among the 3 observers ranged from 45.6% to 93.3% with a mean of 64.9%, while agreement within ±1 point reached 82.2% to 100% (mean: 96.2%). The consistently high within ±1 agreement demonstrates acceptable inter-observer reliability for the ordinal MOS scoring system. At 6 × magnification, CAT-PRSR obtained the highest mean scores across all 6 diagnostic categories – including anatomical visibility, boundary sharpness, microstructure clarity, noise suppression and diagnostic utility – showing no statistically significant differences from the ground truth (*P* > .05). In contrast, RealESRGAN showed moderate performance with statistically significant differences (*P* < .0001), while SwinIR and SeD showed markedly lower scores (*P* < .00001), especially in fine structural visibility and noise control. At 8×, CAT-PRSR maintained perceptual performance within a statistically comparable range to the ground truth for most criteria (*P* > .05), with only minor differences reaching statistical significance (*P* < .01). By contrast, RealESRGAN demonstrated a further decline, with all MOS scores significantly lower than ground truth (*P* < .0001). SwinIR and SeD fell below clinically acceptable thresholds, showing scores near 1.0 and highly significant differences across all categories (*P* < .000001).

## Discussion

This study comprehensively evaluated the proposed CAT-PRSR model for panoramic radiograph super-resolution across multiple magnification levels, demonstrating consistent quantitative and perceptual superiority over existing SR approaches.

The CAT-PRSR model consistently outperformed all comparison models across all magnification levels (4×, 6×, 8×), achieving the highest PSNR/SSIM and lowest LPIPS/FID values (Table 2). Even at extreme 8 × upscaling level, CAT-PRSR maintained sharp, natural images with minimal artifact accumulation, whereas competing CNN-, GAN- and transforme-based models showed severe quality degradation (Figure 2). Expert evaluations confirmed its diagnostic reliability, with MOS scores statistically comparable to ground truth images (*P* > .05). These findings collectively establish CAT-PRSR as a quantitatively and diagnostically reliable SR framework for panoramic radiographs.

SR techniques have evolved from early CNN-based methods to GAN- and transformer-based architectures, expanding their potential in dental panoramic imaging.^1^^,^^3^^,^^5^, ^6^, ^7^^,^^10^^,^^11^^,^^20^^,^^21^ In Stage 2, 3 representative SR models were selected as baselines to represent the main paradigms of contemporary SR research. RealESRGAN^17^ exemplifies a GAN-based approach focusing on perceptual realism and artifact suppression through adversarial optimisation; SwinIR^16^ is a transformer-based model utilising shifted-window self-attention for long-range contextual restoration; and SeD^19^ adopts a hybrid CNN–attention design balancing local refinement and global consistency. These models collectively encompass the principal methodological directions in modern SR development, providing a rigorous basis for comparative evaluation with the proposed CAT-PRSR. Building upon these paradigms, CAT-PRSR integrates confidence-aware feature modulation, enabling simultaneous enhancement of diagnostic visibility and image stability – 2 aspects rarely achieved together in previous SR frameworks. Building upon these paradigms, CAT-PRSR integrates confidence-aware feature modulation, enabling simultaneous enhancement of diagnostic visibility and image stability – 2 aspects rarely achieved together in previous SR frameworks. Unlike conventional super-resolution models that apply uniform loss weighting across all pixels, CAT-PRSR embeds uncertainty estimation directly into the learning objective itself. As a result, the predicted uncertainty actively influences model optimisation by guiding attention toward reliable anatomical structures while suppressing noise-prone or ambiguous regions.

Table 3 summarises how our study aligns with and advances beyond prior super-resolution investigations on dental panoramic radiographs.^1^^,^^3^^,^^5^^,^^6^ Mohammad-Rahimi et al.^1^ reported that the LTE model achieved a PSNR of 39.74 and an SSIM of 0.919, indicating substantial enhancement of fine structural details in panoramic radiographs. Çelik et al.^3^ compared multiple CNN-based SR algorithms and found SRCNN to yield the highest image similarity, with SSIM values ranging from 0.82 to 0.98 across different magnification factors. In a subsequent study, they further demonstrated that SR-enhanced panoramic images significantly improved the classification accuracy of dental structures – such as tooth type identification and periodontal bone level estimation – confirming the clinical applicability and diagnostic relevance of SR technology.^6^ Similarly, Li et al.^5^ conducted a multicentre study involving 608 panoramic images from 2 hospitals and showed that an AI classifier trained on GAN-based SR-enhanced images significantly outperformed the same classifier trained on the original images in predicting mandibular third-molar extraction difficulty, thereby validating the tangible clinical benefit of SR-based enhancement. Collectively, these comparative findings highlight that CAT-PRSR advances beyond prior SR approaches by balancing quantitative fidelity and diagnostic interpretability, a critical aspect contributing to clinical applicability.

**Table 3:** Comparison with super-resolution studies on panoramic radiographs.

Study	SR Models Evaluated	Best Model	Scale	PSNR	SSIM	Other Metrics	Key Findings
Li et al.^5^	GAN + Transfer Learning	GAN-SR	4×	–	–	AUC (diagnosis)	SR improved diagnostic AUC from 0.82 (HR) to 0.96; clinical benefit demonstrated
Mohammad-Rahimi et al.^1^	SRCNN, SRGAN, U-Net, SwinIR, LTE	LTE	4×	39.74	0.92	MSE, MOS (expert-rated)	LTE performed best across both objective and subjective metrics; all DL models outperformed bicubic
Celik et al.^3^	SRCNN, ESPCN, SRGAN, Autoencoder	SRCNN	2×, 4×, 8×	28.74–40.28	0.82–0.98	–	SRCNN yielded highest scores; performance declined with increasing magnification
Celik et al.^6^	ESPCN, SRCNN, ESRT	ESPCN, ESRT	2 ×, 4 ×	31–41	0.82–0.97	FSIM, MS-SSIM, MOS, classification %	SR improved tooth classification accuracy; all SR models outperformed originals, especially at lower magnification
This study (2025)	CAT-PRSR, RealESRGAN, SwinIR, SeD	CAT-PRSR	4×, 6×, 8×	36.41–42.01	0.95–0.99	LPIPS, FID, MOS	CAT-PRSR showed best pixel and diagnostic fidelity at all scales; MOS nearly identical to HR

The proposed CAT-PRSR framework has the potential to substantially advance clinical dental radiology by providing consistently high-quality, artifact-minimised panoramic images across various magnifications. Enhanced image resolution and anatomical fidelity may facilitate earlier and more accurate detection of subtle pathologies – such as incipient caries, early periodontal bone loss and small cystic or neoplastic lesions – thereby reducing diagnostic uncertainty and minimising the need for repeat imaging.^1^^,^^3^^,^^6^^,^^8^^,^^9^ These improvements can directly benefit both clinicians and patients by supporting faster, more confident decision-making in complex or suboptimal imaging conditions, such as in paediatric or medically compromised populations.^6^^,^^9^^,^^22^ Beyond improved visual interpretability, integrating SR-enhanced images into digital workflows could strengthen the performance and reliability of downstream AI-based diagnostic tools.^3^^,^^5^^,^^6^ As dental radiology continues its transition toward fully digital and AI-assisted practice environments, SR frameworks such as CAT-PRSR may help establish a new benchmark for radiographic image quality and diagnostic precision. Ultimately, the integration of SR technology into clinical workflows represents an important advancement toward achieving greater diagnostic consistency, efficiency and patient-centred care in dental radiology.^1^^,^^6^^,^^22^^,^^23^

While our findings are promising, several limitations should be acknowledged. First, the SR models, including CAT-PRSR, were trained and tested on panoramic radiographs from a single institution using limited device models and acquisition protocols, which may restrict generalisability to other manufacturers or imaging conditions. Hence, external validation across diverse equipment is warranted. Second, the transformer-based architecture of CAT-PRSR requires substantial computational resources, making real-time or large-scale deployment challenging in resource-limited clinical environments without high-end GPUs. Third, this study primarily assessed the general image quality and structural fidelity of SR-enhanced panoramic radiographs rather than their impact on specific diagnostic performance. While CAT-PRSR demonstrated strong preservation of anatomical detail and diagnostic interpretability in expert evaluations, we did not directly measure whether its use translates into measurable improvements in diagnostic sensitivity, specificity, or accuracy for particular diseases. For example, although enhanced visualisation of fine structures may theoretically aid in detecting early carious lesions, periapical pathologies, or subtle osseous changes, no dedicated task-based observer study (eg, comparing diagnostic performance with and without SR enhancement) was conducted. Lastly, intraobserver agreement was not assessed in this study. Each examiner evaluated each image only once to minimise recall bias, which precluded assessment of within-examiner consistency over time. Future studies should incorporate repeated assessments with appropriate wash out periods to enable comprehensive evaluation of intraobserver reliability.

To address these limitations, future work will focus on large-scale validation, efficiency optimisation and diagnostic integration. First, a multicentre prospective study will be conducted in collaboration with multiple domestic and international institutions to rigorously assess the model’s generalisability across diverse imaging devices, acquisition protocols and population groups. Second, efforts will be made to develop a lightweight and optimised version of CAT-PRSR that enables real-time SR processing on standard PCs or mobile platforms without dependence on high-end GPUs. Third, disease-specific investigations will aim to quantitatively evaluate how SR enhancement influences diagnostic sensitivity and specificity for various conditions such as caries, periodontal disease, cysts and tumours. Finally, future extensions of the CAT-PRSR framework should explore its application beyond panoramic radiographs – particularly to three-dimensional imaging modalities such as CBCT, CT and MRI – to enhance spatial resolution and improve diagnostic performance across a wider range of clinical contexts.

## Conclusion

The proposed CAT-PRSR framework, built upon a confidence-aware transformer architecture, demonstrated superior performance across all critical dimensions of super-resolution for panoramic radiographs – including quantitative accuracy, structural fidelity, perceptual quality and diagnostic utility. Importantly, it maintained robust performance even at high magnification levels, underscoring its potential to enhance low-resolution dental images and to support more accurate and consistent clinical diagnoses in routine practice.

## Author contribution

Jaehyup Lee: Conceptualization, methodology, software development, data curation, experiment execution, and original manuscript drafting. Young-Eun Kwon: Supervision, clinical validation, result interpretation, and manuscript revision. Chang-Hyeon An and Seo-Young An: Contributed to expert evaluation and validation of diagnostic image quality. Eun-Kyong Kim: Contributed to statistical analysis and quantitative data interpretation.

## Declaration of competing interest

The authors declare that they have no known competing financial interests or personal relationships that could have appeared to influence the work reported in this paper.

## Appendix Supplementary materials

Image, application 1

Image, application 2

Image, application 3

## Footnotes

Supplementary material associated with this article can be found in the online version at doi:10.1016/j.identj.2026.109590.
