# Supplementary material for: Quantitative and Qualitative Evaluation of a Confidence-Aware Transformer-Based Super-Resolution Framework for Panoramic Radiographs
Source: Int Dent J. 2026 Apr 27;76(4):109590. doi: 10.1016/j.identj.2026.109590 (PMC13137014; doi:10.1016/j.identj.2026.109590)
Supplement: Supplementary file 1 [file mmc1.docx]

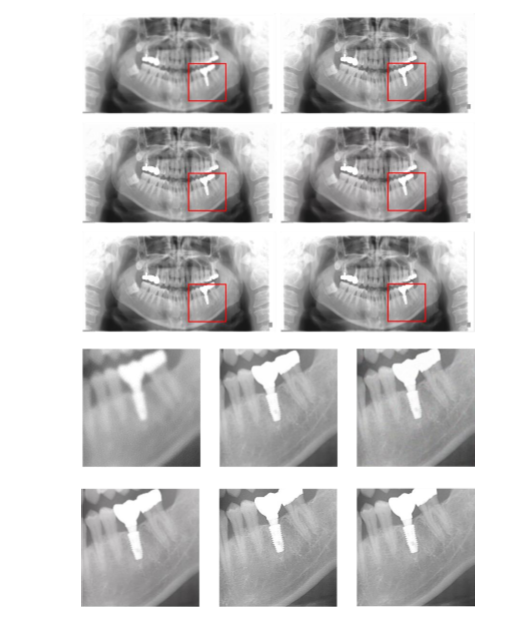


**Supplementary FigureS1. Qualitative comparison of SR models on panoramic radiographs.(Stage 1)**(A) Original low-resolution input, (B) RealESRGAN image, (C) SeD image, (D) SwinIR image, (E) CAT-PRSR image, (F) Ground truth image, (G-L) Zoomed views of the red boxed region corresponding to (A–F), respectively.
