# Supplementary material for: Quantitative and Qualitative Evaluation of a Confidence-Aware Transformer-Based Super-Resolution Framework for Panoramic Radiographs
Source: Int Dent J. 2026 Apr 27;76(4):109590. doi: 10.1016/j.identj.2026.109590 (PMC13137014; doi:10.1016/j.identj.2026.109590)
Supplement: Supplementary file 2 [file mmc2.docx]

Table S1. Architectural details of the CAT-PRSR backbone network

| **Component** | **Details** |
| --- | --- |
| RSTBs | 4 blocks |
| STLs per RSTB | 6 (total 24 STLs) |
| Window size | 8×8 |
| Embed dim | 180 |
| Attention heads | [6, 6, 6, 6] |
| Total parameters | 8.07 M |
