# Supplementary material for: Quantitative and Qualitative Evaluation of a Confidence-Aware Transformer-Based Super-Resolution Framework for Panoramic Radiographs
Source: Int Dent J. 2026 Apr 27;76(4):109590. doi: 10.1016/j.identj.2026.109590 (PMC13137014; doi:10.1016/j.identj.2026.109590)
Supplement: Supplementary file 3 [file mmc3.docx]

| **Model** | **Observer** | **MOS_Overall** | **Anatomy_Visibility** | **Boundary**  **_Sharpness** | **Microstructure_**  **Visibility** | **Noise_Artifacts** | **Diagnostic_Utility** |
| --- | --- | --- | --- | --- | --- | --- | --- |
| Ground Truth | obs3_ | 4.57 ± 0.57 | 4.90 ± 0.31 | 4.90 ± 0.31 | 4.50 ± 0.51 | 4.53 ± 0.63 | 4.77 ± 0.50 |
| Ground Truth | obs2_ | 4.87 ± 0.35 | 4.93 ± 0.25 | 5.00 ± 0.00 | 4.80 ± 0.41 | 4.87 ± 0.35 | 4.93 ± 0.25 |
| Ground Truth | obs1_ | 4.80 ± 0.41 | 5.00 ± 0.00 | 5.00 ± 0.00 | 4.63 ± 0.49 | 4.97 ± 0.18 | 4.87 ± 0.35 |
| RealESRGAN | obs3_ | 3.57 ± 0.57 (***) | 3.83 ± 0.46 (***) | 3.20 ± 0.55 (***) | 3.17 ± 0.53 (***) | 3.83 ± 0.79 (***) | 3.57 ± 0.57 (***) |
| RealESRGAN | obs2_ | 3.63 ± 0.61 (***) | 3.87 ± 0.51 (***) | 3.33 ± 0.48 (***) | 3.33 ± 0.61 (***) | 3.73 ± 0.69 (***) | 3.50 ± 0.63 (***) |
| RealESRGAN | obs1_ | 3.77 ± 0.57 (***) | 3.83 ± 0.53 (***) | 3.23 ± 0.43 (***) | 3.27 ± 0.58 (***) | 3.77 ± 0.63 (***) | 3.63 ± 0.61 (***) |
| SwinIR | obs3_ | 2.43 ± 0.57 (***) | 2.67 ± 0.48 (***) | 1.93 ± 0.37 (***) | 2.10 ± 0.55 (***) | 2.13 ± 0.51 (***) | 2.60 ± 0.56 (***) |
| SwinIR | obs2_ | 2.03 ± 0.49 (***) | 2.40 ± 0.50 (***) | 1.90 ± 0.31 (***) | 1.80 ± 0.41 (***) | 1.90 ± 0.48 (***) | 2.20 ± 0.61 (***) |
| SwinIR | obs1_ | 2.13 ± 0.35 (***) | 2.47 ± 0.51 (***) | 1.80 ± 0.41 (***) | 1.77 ± 0.57 (***) | 1.80 ± 0.61 (***) | 2.23 ± 0.43 (***) |
| SeD | obs3_ | 2.00 ± 0.37 (***) | 2.43 ± 0.50 (***) | 2.03 ± 0.56 (***) | 1.90 ± 0.55 (***) | 1.80 ± 0.48 (***) | 2.50 ± 0.51 (***) |
| SeD | obs2_ | 2.07 ± 0.25 (***) | 2.40 ± 0.50 (***) | 2.17 ± 0.38 (***) | 1.97 ± 0.41 (***) | 1.77 ± 0.43 (***) | 2.73 ± 0.45 (***) |
| SeD | obs1_ | 2.03 ± 0.41 (***) | 2.57 ± 0.50 (***) | 2.27 ± 0.58 (***) | 1.87 ± 0.43 (***) | 1.63 ± 0.49 (***) | 2.57 ± 0.50 (***) |
| CAT-PRSR | obs3_ | 4.57 ± 0.50 (ns) | 4.73 ± 0.45 (ns) | 4.70 ± 0.47 (ns) | 4.40 ± 0.62 (ns) | 4.37 ± 0.67 (ns) | 4.67 ± 0.48 (ns) |
| CAT-PRSR | obs2_ | 4.63 ± 0.49 (*) | 4.87 ± 0.35 (ns) | 4.67 ± 0.48 (***) | 4.43 ± 0.63 (**) | 4.47 ± 0.51 (**) | 4.93 ± 0.25 (ns) |
| CAT-PRSR | obs1_ | 4.73 ± 0.45 (ns) | 4.77 ± 0.43 (**) | 4.70 ± 0.47 (**) | 4.53 ± 0.51 (ns) | 4.37 ± 0.61 (***) | 4.83 ± 0.38 (ns) |

**Supplementary Table S2**

(A) Mean Opinion Scores (MOS) for 6× Magnification among Ground Truth and Four Super-Resolution Models.

(B) Mean Opinion Scores (MOS) for 8× Magnification among Ground Truth and Four Super-Resolution Models

| **Model** | **Observer** | **MOS_Overall** | **Anatomy_Visibility** | **Boundary**  **_Sharpness** | **Microstructure_**  **Visibility** | **Noise_Artifacts** | **Diagnostic_Utility** |
| --- | --- | --- | --- | --- | --- | --- | --- |
| Ground Truth | obs3_ | 4.57 ± 0.57 | 4.90 ± 0.31 | 4.90 ± 0.31 | 4.43 ± 0.57 | 4.53 ± 0.63 | 4.77 ± 0.50 |
| Ground Truth | obs2_ | 4.33 ± 0.61 | 4.80 ± 0.41 | 4.83 ± 0.38 | 4.40 ± 0.50 | 4.30 ± 0.70 | 4.63 ± 0.61 |
| Ground Truth | obs1 | 4.93 ± 0.25 | 5.00 ± 0.00 | 5.00 ± 0.00 | 4.73 ± 0.45 | 4.67 ± 0.48 | 4.93 ± 0.25 |
| RealESRGAN | obs3_ | 2.63 ± 0.49 (***) | 3.57 ± 0.57 (***) | 2.63 ± 0.49 (***) | 2.53 ± 0.68 (***) | 4.03 ± 0.81 (*) | 2.50 ± 0.51 (***) |
| RealESRGAN | obs2_ | 2.67 ± 0.48 (***) | 3.63 ± 0.49 (***) | 2.73 ± 0.45 (***) | 2.67 ± 0.66 (***) | 3.77 ± 0.77 (**) | 2.67 ± 0.48 (***) |
| RealESRGAN | obs1 | 2.30 ± 0.47 (***) | 3.17 ± 0.70 (***) | 2.60 ± 0.50 (***) | 2.37 ± 0.56 (***) | 3.83 ± 0.70 (***) | 2.27 ± 0.45 (***) |
| SwinIR | obs3_ | 1.63 ± 0.49 (***) | 2.57 ± 0.57 (***) | 1.63 ± 0.49 (***) | 1.53 ± 0.68 (***) | 1.67 ± 0.71 (***) | 2.43 ± 0.63 (***) |
| SwinIR | obs2_ | 1.23 ± 0.43 (***) | 2.37 ± 0.67 (***) | 1.47 ± 0.51 (***) | 1.27 ± 0.45 (***) | 1.37 ± 0.72 (***) | 2.13 ± 0.57 (***) |
| SwinIR | obs1 | 1.43 ± 0.50 (***) | 2.43 ± 0.50 (***) | 1.60 ± 0.50 (***) | 1.33 ± 0.61 (***) | 1.60 ± 0.77 (***) | 2.37 ± 0.49 (***) |
| SeD | obs3_ | 1.07 ± 0.25 (***) | 1.43 ± 0.50 (***) | 1.03 ± 0.18 (***) | 1.03 ± 0.18 (***) | 1.07 ± 0.25 (***) | 1.47 ± 0.51 (***) |
| SeD | obs2_ | 1.07 ± 0.25 (***) | 1.43 ± 0.50 (***) | 1.07 ± 0.25 (***) | 1.00 ± 0.00 (***) | 1.13 ± 0.35 (***) | 1.47 ± 0.51 (***) |
| SeD | obs1 | 1.00 ± 0.00 (***) | 1.37 ± 0.49 (***) | 1.00 ± 0.00 (***) | 1.10 ± 0.31 (***) | 1.20 ± 0.41 (***) | 1.73 ± 0.45 (***) |
| CAT-PRSR | obs3_ | 4.37 ± 0.61 (ns) | 4.50 ± 0.51 (**) | 4.40 ± 0.62 (***) | 4.40 ± 0.62 (ns) | 4.30 ± 0.65 (ns) | 4.40 ± 0.62 (*) |
| CAT-PRSR | obs2_ | 4.30 ± 0.60 (ns) | 4.47 ± 0.51 (**) | 4.37 ± 0.61 (**) | 4.20 ± 0.66 (ns) | 4.30 ± 0.65 (ns) | 4.30 ± 0.60 (*) |
| CAT-PRSR | obs1 | 4.13 ± 0.51 (***) | 4.40 ± 0.50 (***) | 4.30 ± 0.60 (***) | 4.20 ± 0.41 (***) | 4.27 ± 0.74 (**) | 4.13 ± 0.51 (***) |
